# Bilateral hypoplasia of the internal carotid artery, presenting as a subarachnoid hemorrhage secondary to intracranial aneurysmal formation: a case report

**DOI:** 10.1186/1752-1947-6-45

**Published:** 2012-01-30

**Authors:** Arshad A Siddiqui, Zain A Sobani

**Affiliations:** 1 Pakar Neurosurgery, Hospital Sultanah Aminah, Jalan Abu Bakar, Johor Bahru-80100, Johor-Malaysia; 2 Section of Neurosurgery, Department of Surgery, Aga Khan University, Stadium Road, Karachi, Pakistan

## Abstract

**Introduction:**

Hypoplasia of the internal carotid artery is a rare congenital anomaly, with only 24 cases of bilateral internal carotid artery hypoplasia reported to date. Here, we present the case of a 48-year-old woman with bilateral internal carotid artery hypoplasia. She had a collateral circulation through the vertebrobasilar system; however given the high pressure flow she developed aneurysmal formations in the posterior communicating artery. To the best of our knowledge, only seven reported cases of internal carotid artery hypoplasia have been associated with intracranial aneurysmal formations.

**Case presentation:**

A 48-year-old Sindhi woman from Karachi, Pakistan, presented to our emergency room with a sudden onset headache and was diagnosed as having a subarachnoid hemorrhage. Digital subtraction angiography revealed hypoplasia of her internal carotid artery bilaterally with an associated fusiform aneurysm of the posterior communicating artery. Our patient declined any operative intervention in view of the associated risks. She died of a rebleed six weeks after her initial presentation.

**Conclusion:**

Bilateral internal carotid artery occlusions can present with subarachnoid hemorrhages due to associated intracranial aneurysm formation. Prior knowledge about the possible existence of such angioarchitectural arrangement is mandatory for an early diagnosis. However, even with prompt diagnosis, management options with acceptable risk-benefit equations are still unavailable.

## Introduction

Hypoplasia of the internal carotid artery (ICA) is a rare congenital anomaly, usually noticed as a narrowing in the lumen of the artery 1 cm to 2 cm above its bifurcation [1-4]. About 60 cases of ICA hypoplasia have been reported in the literature to date, of which 24 cases have been bilateral. Here we report the case of a 48-year-old woman with bilateral ICA hypoplasia, presenting with a subarachnoid hemorrhage secondary to an aneurysm of the posterior communicating artery. To the best of our knowledge only seven reported cases of ICA hypoplasia have been associated with intracranial aneurysmal formations [1,5-10]. We review the common clinical manifestations, diagnostic modalities and clinicopathological data while comparing the reported cases with our case.

## Case presentation

A 48-year-old Sindhi woman, from a middle class household of Karachi, Pakistan, presented to our clinic with sudden onset of loss of consciousness followed by severe headache and nausea. There was no history of trauma, fever, fits or blurring of vision. Her past medical history did not reveal any comorbid medical conditions and she had not had any similar episodes in the past.

On examination she was drowsy. She had a pulse of 90 beats per minute, her blood pressure was 165/90 mmHg and her temperature was 37.5°C. Her Glasgow Coma Scale was 14 out of 15. Both her pupils were equal and reactive to light. A fundoscopic examination was within normal limits. She had mild neck rigidity but no other signs of meningeal irritation. There were no other neurological deficits. She was clinically diagnosed with a subarachnoid hemorrhage with Hunt and Hess grade 2.

**Figure 1:**
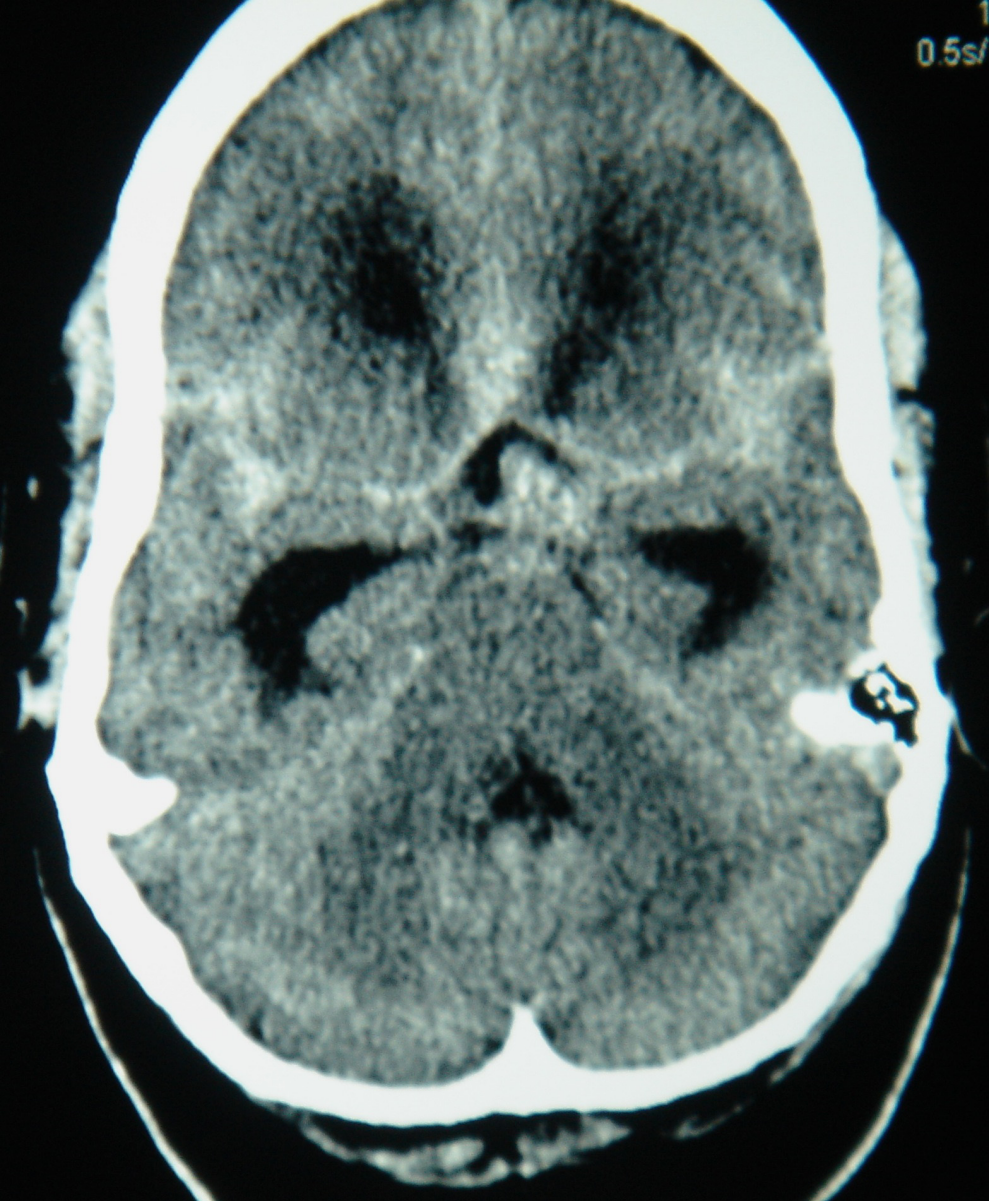
**Unenhanced computed tomography scan of her brain showing diffuse subarachnoid hemorrhage in the basal cisterns**. Abnormal hyperdense signals in the prepontine cistern indicate an underlying vascular lesion.

Her baseline hematological work-up was normal except for a raised total leukocyte count of 16,000 cells/microliter. A computerized tomography scan showed diffuse hemorrhage in her subarachnoid spaces with a small area of focal hyperdensity in the prepontine area, raising suspicion of an aneurysm of the basilar artery (Figure 1).

**Figure 2:**
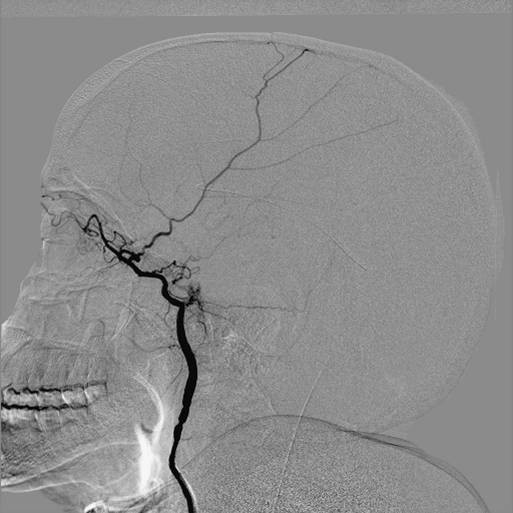
**Selective injection in the left internal carotid artery on digital subtraction angiography showing the internal carotid artery terminating into the ophthalmic artery with no contribution to cerebral vasculature**.

**Figure 3:**
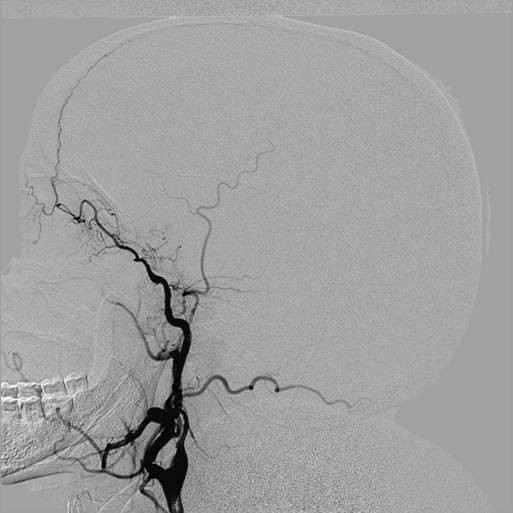
**Selective injection in the right common carotid artery on digital subtraction angiography showing exclusive termination of the right internal carotid artery into the ophthalmic artery with no contribution to cerebral circulation**. The external carotid artery is also not providing any collateral contribution to the cerebral vascular supply.

**Figure 4:**
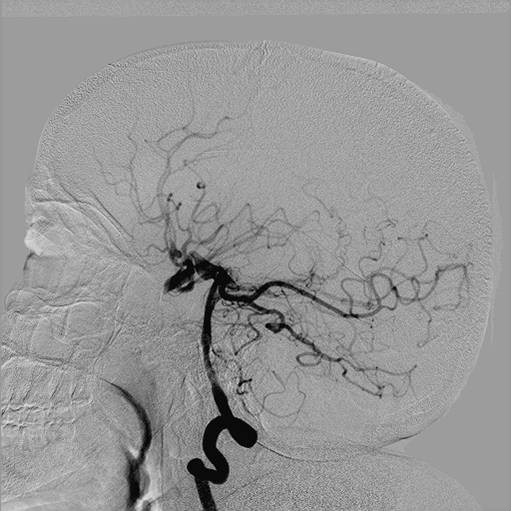
**Selective injection of the right vertebral artery showing an enlarged posterior communicating artery opacifying the supra-ophthalmic segments of the internal carotid artery with exclusive contribution to the anterior circulation bilaterally**. There is fusiform dilatation of the right posterior communicating artery at the basilar end, suggestive of a fusiform aneurysm of the right posterior communicating artery.

**Figure 5:**
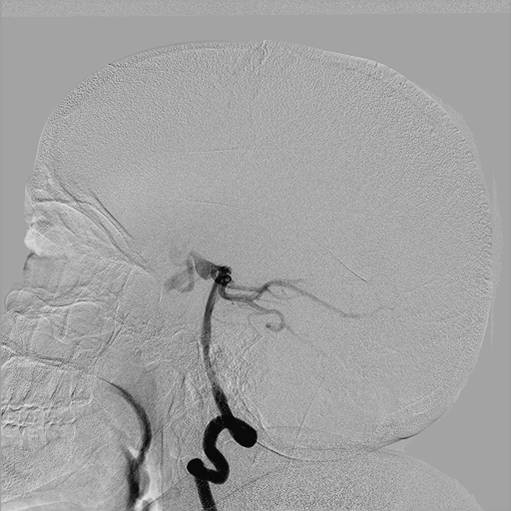
**Early arterial phase of the right vertebral artery injection, delineating the configuration of a fusiform aneurysm at the basilar end of the right posterior communicating artery**. It appears that the origin of the right P1 segment of the posterior cerebral artery is incorporated in the aneurysm segment and the posterior communicating artery distal to the aneurysm is obtuse. The ipsilateral supraclinoidal artery is also perfused by this injection.

**Figure 6:**
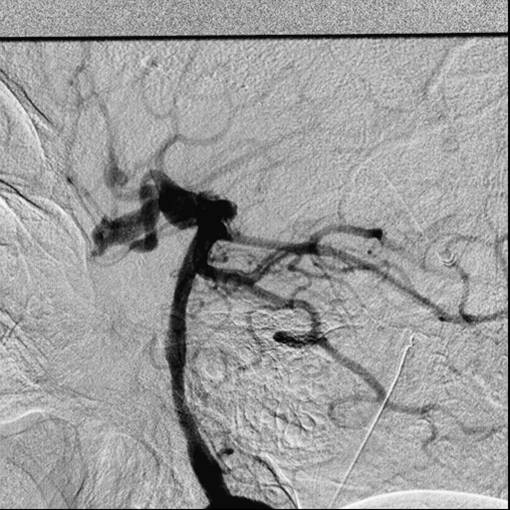
**Magnified view of the right vertebral injection highlighting the anatomical details described in Figures 4 and 5**.

Digital subtraction angiography showed a normal origin of the common carotid and vertebral arteries; however, both ICAs were hypoplastic from 1 cm to 2 cm above the carotid bifurcation. The vessels ended in her cranial cavity after feeding her ophthalmic arteries. Her anterior and middle cerebral arteries could not be visualized on the angiograms (Figures 2,3 and 4); however, her external carotid arteries and their branches were normal. A selective angiogram of her vertebral arteries showed collaterals to her anterior and middle cerebral arteries on both sides through enlarged posterior communicating arteries. A fusiform aneurysm of the right posterior communicating artery was also noted at its basilar end (Figures 4, 5 and 6).

Subsequent management via microsurgical techniques was proposed to our patient. However, considering the absence of major symptoms and the increased risk of surgery, she refused any therapeutic procedure. Unfortunately, our patient died of a rebleed six weeks after her initial presentation.

## Discussion

Hyrtle was the first to describe the presence of hypoplasia of the ICA in 1836 in a postmortem series [11]. Since then, about 60 cases of ICA hypoplasia have been described in the literature. However, the first cases of bilateral ICA hypoplasia in living patients were reported by Hawkins and Scott in 1967 [12] and there have since been a total of 24 reported cases.

It has been hypothesized that hypoplasia of the ICA arises due to the incomplete development of the fetal dorsal aorta, which normally gives rise to the distal cervical segment of the ICA, up to the clinoidal segment [13]. This theory is also supported by the relatively constant location of the hypoplastic segment (that is, 1 cm to 2 cm above the bifurcation to the cranium) and the fact that the first cervical segment, which develops from the third aortic arch, is normal.

The hypoplastic arteries may either continue intracranially or become occluded at some intracranial point; often they lead into the ophthalmic arteries [10,14]. In our case, the hypoplastic arteries also terminated as the ophthalmic arteries bilaterally.

In cases of bilateral hypoplasia of the ICA, the blood supply to the intracranial carotid systems may develop by one of three different mechanisms [11-13]. Most frequently, a collateral circulation is seen to arise from the vertebrobasilar system through enlarged posterior communicating arteries [7]. This was seen in 18 of the 24 reported cases. Less frequently, the anterior circulation is supplied by branches of the external carotid, internal maxillary and ophthalmic arteries through a carotid rete mirabile at the base of the brain [8,12], or through pseudoangiomatous anastomoses of the basal ganglia and thalamus, supplied by the posterior cerebral and posterior choroidal arteries [7].

The actual prevalence of this condition is unknown. A large study reviewing 5,100 magnetic resonance imaging scans and traditional angiograms found seven patients with congenital absence or hypoplasia of the ICA. Further, they found only one case of bilateral ICA hypoplasia in their sample [15].

The clinical presentation of patients with bilateral hypoplasia was usually sudden, attributable mainly to hemodynamic insufficiency states or hemorrhagic episodes. The average age at presentation was 36 years (range 17 years to 64 years). Seven patients presented with aneurysmal subarachnoid hemorrhage. Presentation with an epileptic seizure has also been described in three cases [7,10,16]. This may be attributed to decreased perfusion of the cerebral hemispheres.

In our review of the literature, we found that collateral circulation most commonly arises from the vertebrobasilar system, therefore it comes as no surprise that the most frequent location of aneurysms are the basilar and posterior cerebral arteries. The direction of the aneurysm sac (as in our case) confirms that the aneurysm formation was induced by hemodynamic modifications in the vertebrobasilar system.

Saccular aneurysms associated with carotid artery hypoplasia, as with other arterial anomalies, have a high tendency to bleed because of the increased blood flow in the parent arterial system. Thus, endovascular stent placement or surgical clipping is mandatory, particularly in patients with previous bleeding. However, given the parent malformation, these patients are at a higher risk of complications related to either modality of intervention. Unfortunately, our patient had a fusiform aneurysm, which limited the therapeutic options available to her.

If we were to consider an endovascular procedure in order to place an intramural stent, access to the saccular segment of her posterior communicating artery would be a technical challenge owing to the longer navigation route and its impact on the stability of catheter guidance systems currently available. The Novel SILK stent system can be a favorable option in this regard [17]. However, delivery and stability demands a high level of technical expertise and may involve a major risk of thromboembolic complications during and after the procedure. Further, the obtuse angle of her right posterior communicating artery distal to the aneurysm posed technical difficulties, limiting the stability of the intramural stent system. Stent-assisted coiling of this aneurysm would also not have been feasible due to the peculiar angioarchitecture, aneurysm morphology and a lack of technical tools along with the high cost of the procedure.

Therefore, our patient was offered microsurgical management, which she declined. Of the seven reported cases of bilateral carotid hypoplasia, only one underwent successful clipping of the aneurysm [18], whereas six refused treatment when informed about the risks of intervention.

## Conclusion

Bilateral ICA occlusions presenting with subarachnoid hemorrhage due to associated intracranial aneurysm are an extremely rare clinical entity. Prior knowledge about the possible existence of such angioarchitectural arrangement will prompt early diagnosis. Management with acceptable operative risks is still a challenge and rebleeding remains a major killer in the natural course of the disease. An interventional procedure with an optimal risk-benefit equation is yet to be determined for these patients.

## Consent

Written informed consent was obtained from the patient for publication of this case report and accompanying images. This was done in the presence of the patient's next of kin. A copy of the written consent is available for review by the Editor-in-Chief of this journal.

## Competing interests

The authors declare that they have no competing interests.

## Authors' contributions

Both ZAS and AAS were involved in the primary care of the patient. ZAS analyzed and interpreted the patient data. AAS was a major contributor to the draft manuscript, which was later edited by ZAS. Both authors read and approved the final manuscript.
